# Probing the current-phase relation in Josephson point-contact junctions between $\hbox {Pb}_{0.6}$In$_{0.4}$ and $\hbox {Ba}_{0.6}$K$_{0.4}$(FeAs)$_2$ superconductors

**DOI:** 10.1038/s41598-021-00762-0

**Published:** 2021-12-14

**Authors:** Valeriy A. Stepanov, Chengtian Lin, Renato S. Gonnelli, Mauro Tortello

**Affiliations:** 1 P.N. Lebedev Physical Institute of the Russian Academy of Sciences, Moscow, Russia 119333; 2 Max-Planck-Institut für Festkörperforschung, 70569 Stuttgart, Germany; 3 Dipartimento di Scienza Applicata e Tecnologia, Politecnico di Torino, Torino, 10129 Italy

**Keywords:** Superconducting properties and materials, Superconducting properties and materials

## Abstract

The Josephson effect in point contacts between an “ordinary” superconductor $\hbox {Pb}_{0.6}$In$_{0.4}$ ($T_c \approx 6.6 \, {\mathrm {K}}$) and single crystals of the Fe-based superconductor Ba$_{0.6}$K$_{0.4}$(FeAs)$_2$ ($T_c \approx 38.5 \, {\mathrm {K}}$), was investigated. In order to shed light on the order parameter symmetry of Ba$_{0.6}$K$_{0.4}$(FeAs)$_2$, the dependence of the Josephson supercurrent $I_s$ on the temperature and on $\sin (d\varphi )$ with $d = 1, 2$ was studied. The dependencies of the critical current on temperature $I_c (T)$ and of the amplitudes of the first current steps of the current–voltage characteristic $i_n^{exp} (\sqrt{P})$ $(n = 0, 1, 2)$ on the power of microwave radiation with frequency $f = (1.5 \div 8)\, {\mathrm {GHz}}$ were measured. It is shown that the dependencies $I_c (T)$ are close to the well-known Ambegaokar–Baratoff (AB) dependence for tunnel contacts between “ordinary” superconductors and to the dependence calculated by Burmistrova et al. (Phys Rev B **91**, 214501 (2015)) for microshorts between an “ordinary” superconductor and a two-band superconductor with $s \pm$ order parameter symmetry at certain values of the transparency of boundaries and thickness of the transition layer. It is found that the dependencies $i_n^{exp} (\sqrt{P})$ cannot be approximated within the resistively shunted model using the normalized microwave frequencies $\Omega = 2 \pi f / (2eV_c / \hbar )$ with characteristic voltages $V_c = I_c R_N$, $(R_N$—normal resistance of the contact) found from the low-voltage parts of the current–voltage characteristics. The reasons for this failure are discussed and a method is proposed for accurately determining the value of $\Omega$, which takes into account all the features of the point contact affecting the period of the dependence $i_n^{exp} (\sqrt{P})$. An analysis of the $I_c (T)$ and $i_n^{exp} (\sqrt{P})$ dependencies shows that the superconducting current of the Josephson contacts under investigation is proportional to the $\sin$ of the phase difference $\varphi$, $I_s = I_c sin(\varphi )$. The implications of these results on the symmetry of the order parameter are also discussed.

## Introduction

Despite the presence of many studies on multiband layered Fe-based superconductors (FeBS), discovered more than 10 years ago^1^, the interaction leading to the formation of the pairs has not yet been, up to now, unambiguously proven. The majority of the scientific community considers, however, that the most plausible scenario for the appearance of high-temperature superconductivity in these compounds is an electronic interband pairing based on spin fluctuations^2^. The appearance of this type of interaction is related to the shape and arrangement of the several hole and electron pockets that form their Fermi surface. In this case, the relatively weak superconductivity caused by the electron-phonon interaction in individual pockets with the usual *s*-wave symmetry of the order parameter (OP) is strongly enhanced by the interband spin fluctuations, that lead to the so-called $s \pm$-wave symmetry of the OP^2,3^. It is an isotropic singlet pairing that features different signs of the OP in the electronic, $s-$ and hole, $s+$ pockets. In an ideal two-band superconductor with $s \pm$ symmetry, the OP phases in the $s -$ and $s +$ pockets should differ by $\pi$. However, in real Fe-based superconductors up to five bands are generally involved in the $s \pm$-wave superconducting coupling: in such systems, the time reversal symmetry can be violated and the phase difference between the OP of different pockets can be arbitrary^4^. Theoretical studies of the two-band model of a superconductor with an $s \pm$ symmetry of the OP showed that such a symmetry should affect a number of characteristics of the Josephson superconducting current, $I_s$ between an “ordinary” , superconductor *S* and a two-band one. Indeed, in this case, the *S*/FeBS Josephson junction (JJ) consists of two contacts, a “normal” one, $S/s+$ with $I_s^+$ current, and the so-called $\pi$-contact, $S/s-$^4^ with the $I_s^-$ current that has opposite direction with respect to $I_s^+$. The interference of the supercurrents directed against each other leads to (1) the suppression of the total JJ current $I_s=I_s^+ - I_s^-$, (2) a decrease in the critical current $I_c$ of the contact, (3) a change in the temperature dependence of $I_c$ and (4) a proportionality of $I_s$, under certain conditions, to $\sin (2 \varphi )$^5–15^. This fact gives a strong support to the idea of trying to determine the OP symmetry in Fe-based superconductors, and the corresponding pairing interaction, by studying the Josephson effect in these compounds.

A series of experimental works has been devoted to the study of the supercurrent in point-contact (PC) and planar Josephson junctions of the *S*/FeBS type^16–25^, which revealed the main features of the Josephson effect in those structures. However, with the exception of Refs.^15,24,25^, the current-phase relationship was not evaluated. Recent theoretical studies of the supercurrent $I_s$ in *S*/FeBS JJ in the framework of the two-band FeBS model showed that, for the $s \pm$ symmetry of the order parameter, the supercurrent flowing along the $c-$axis of FeBS is proportional to $\sin (\varphi )$ as in ordinary *S*/*S* junctions, whereas it is expected to be proportional to $\sin (2 \varphi )$ along the *ab* plane under certain conditions^15^. Also some experimental studies of the pair current of JJ between Pb$_{0.7}$In$_{0.3}$ and overdoped Ba$_{0.4}$K$_{0.6}$(FeAs)$_2$ ($T_c \approx 30 \, {\mathrm {K}}$) qualitatively confirmed this conclusion^15,24^. A particular interest in this type of experiments arises from the fact that it is possible to distinguish between the two types of current-phase relationships, $I_s \propto \sin (\varphi )$ or $I_s \propto \sin (2 \varphi )$, by performing relatively simple experiments that do not require the creation of multi-contact Josephson structures. For doing these experiments, it is just necessary to study the oscillations, that is, the changes from the maximum to the minimum value, of the amplitude of the critical current and of the first few Shapiro steps, $I_n$ ($n=0,1,2$), that appear in the current–voltage characteristic of a JJ under electromagnetic microwave irradiation, as a function of the radiation power, *P*^15,24,25^. The measured dependence $i_n^{exp} (\sqrt{P}) = I_n (\sqrt{P}) / I_c (P = 0)$ can be compared to the calculated one $i_n^{calc} (i_{ac})$ ($i_{ac} \propto \sqrt{P}$ is the current induced by the microwave signal in the JJ) by using the resistively shunted junction model (RSJ) of the JJ. The period of the measured oscillations $i_n^{exp} (\sqrt{P})$ within this model is uniquely determined by the normalized frequency of the external microwave signal $\Omega =2 \pi f/(2e I_c R_N / \hbar )$, where *f* is the microwave frequency and $R_N$ is the junction resistance in the normal state, and by the dependence of $I_s$ on the phase difference of the OP in the banks of the contact^26^. It is well known that the RSJ model leads to noticeably different oscillation periods of the current steps in the $I-V$ characteristics in case of $I_s \propto \sin (\varphi )$ or $I_s \propto \sin (2 \varphi )$, which, in principle, should make it obvious to determine the $I_s(\varphi )$ dependence. However, as some theoretical studies have reported, in JJs between an ordinary superconductor and the simplest, two-band FeBS with an $s \pm$ symmetry of the order parameter, the current steps with certain indices *n* can be weakened and even disappear^10,11^. Furthermore, when taking into account a larger number of conductive bands in the FeBS, the description of the characteristic current steps becomes even more complicated^5^. In this case, the oscillation periods and amplitudes of the current steps are determined by the difference of the order parameter phase between different bands of the FeBS, and by the interference of the supercurrents in more than two channels. Therefore, to unambiguously determine the current-phase relationship $I_s(\varphi )$, it is necessary to make an exact comparison between the oscillation periods in the experimental $i_n^{exp}(\sqrt{P})$ and in the one calculated from the RSJ model, $i_n^{calc}(i_{ac})$. The theoretical oscillation periods have to be determined from the curves obtained by using the RSJ model solved, obviously, for both $I_s \propto \sin (\varphi )$ and $I_s \propto \sin (2\varphi )$.

In this work, we studied the supercurrent characteristics of Josephson point contacts between an “ordinary” Pb$_{1-x}$In$_x$ superconductor ($x=0.4$, $T_c \approx 6.6 \, {\mathrm {K}}$) and optimally doped Ba$_{1-x}$K$_{x}$(FeAs)$_2$ ($x=0.4$, $T_c \approx 38.5 \, {\mathrm {K}}$) with current injection mainly along the *ab* plane, even though injection along the *c*-axis was investigated as well. We have studied the dependencies of critical currents on temperature and the dependence of the critical currents and amplitudes of the first current steps of a number of contacts on the power of electromagnetic microwave radiation *P* with frequencies *f* = $(1.5 \div 8) \,{\mathrm {GHz}}$ at temperatures in the range ($1.8 \div 4.2)\, {\mathrm {K}}$.

## Experimental results

**Figure 1:**
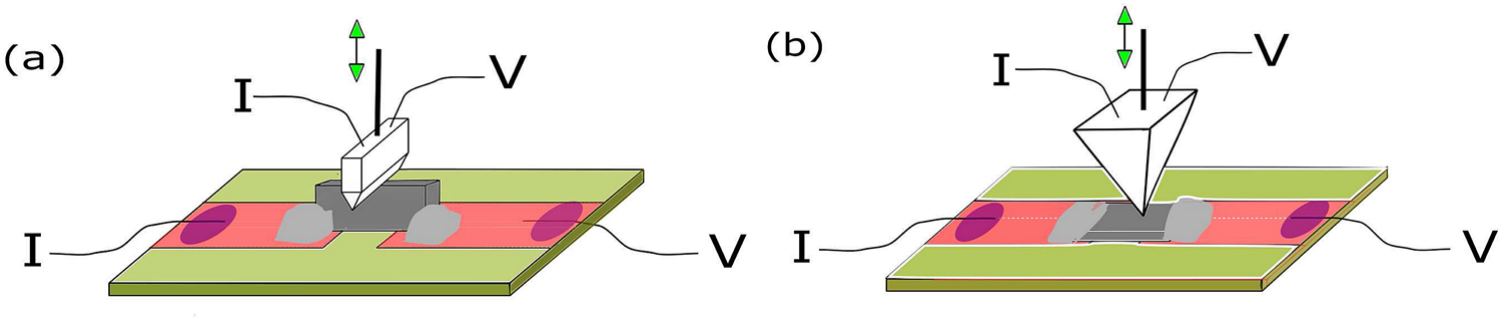
Schemes of the point-contacts forming the four-probe electric connections for $I-V$ measurements in a microwave field *P*. FeBS single-crystal plate was clamped by In on a foiled glass-fiber plastic substrate. The movable PbIn *S* electrode was soldered to copper wire and was pressed to the FeBS plate. A probe current *I* is injected into the PC and the voltage drop *V* across the contact is measured. The microwave signal was supplied by a cable. (**a**) Scheme of the PC with current injection along *ab* plane. (**b**) PC with current injection along *c* axis.

The properties of a JJ are reflected in its current-voltage characteristics (CVC). To record the $I-V$ characteristic, as a rule, the dc-current *I* through the contact is set by using a current source and the voltage drop *V* across it is measured. In this work, the study of the Josephson effect between an “ordinary” superconductor Pb$_{1-x}$In$_x$ and a Ba$_{0.6}$K$_{0.4}$(FeAs)$_2$ single crystal was carried out by using point contacts. A scheme of the point contacts with current injection along the *ab* plane and the *c* axis is shown in Fig. 1a and b, respectively. The device for creating the PC consisted of a cryogenic insert with a reducer and a differential screw for moving the *S* electrode (accuracy of about $10 \, \mu$m per turn of the adjusting screw) and a fixed base for mounting the FeBS crystal.

The microwave (MW) signal was fed to the contact by a cable with an antenna at the end. After cleaning their surfaces, the *S* electrode ${\hbox {Pb}_{0.6}\hbox {In}_{0.4}}$ and single crystal $\hbox {Ba}_{0.6}\hbox {K}_{0.4}\hbox {(FeAs)}_2$ were installed in a cryogenic insert at room temperature at a distance of $\approx (0.1 \div 0.2) \, {\mathrm {mm}}$ from each other and the device prepared for the measurements was placed in a cryostat with liquid helium. The PC was tuned by moving the *S*-electrode at $T = 4.2 \,{\mathrm {K}}$. The Josephson nature of the pair current was checked by the presence of current steps in the $I-V$ characteristic of the contact when it was irradiated by microwave electromagnetic radiation.

**Figure 2:**
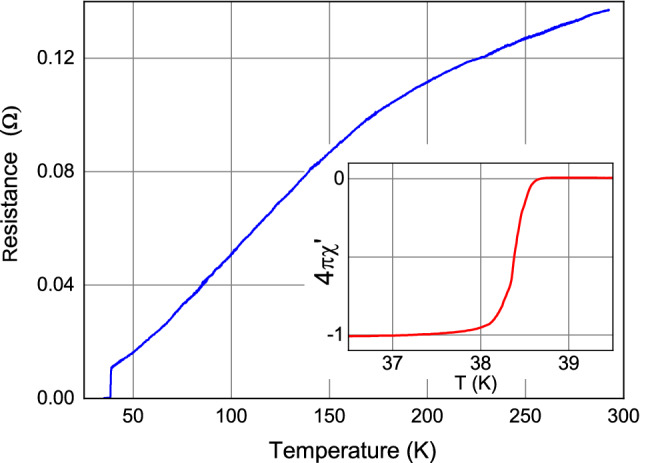
Temperature dependence of the longitudinal resistance $R_{ab}$ of the Ba$_{0.6}$K$_{0.4}$(FeAs)$_2$ single crystal in the temperature range $(4.2 \div 280)\, {\mathrm {K}}$. The inset shows the temperature dependence of the magnetic susceptibility of this sample under cooling in zero magnetic field. $R(300 \, {\mathrm {K}})/R(40 \, {\mathrm {K}}) = 12$, $T_c = 38.5 \, {\mathrm {K}}$, $\Delta T_c (10\% \div 90\%) = 0.3 \, {\mathrm {K}}$ as determined from the magnetic susceptibility.

The characteristics of the superconducting transition of the Ba$_{0.6}$K$_{0.4}$(FeAs)$_2$ crystal used in this work are shown in Fig. 2. The quality of this crystal corresponds to that of the best known samples^27–33^. All measurements presented in this work were made on this crystal.

No superconducting current was usually observed at the first touch between the two electrodes, due to the possible presence of oxides on their surface. However, when increasing the pressure, the contact resistance decreased and a superconducting current $I_c$ arised, whose value increased with increasing pressure. When the junction was irradiated by microwaves, the point-contact critical current $I_c$ decreased and current steps appeared in the $I-V$ curve at a contact voltage $V_n=n \hbar \omega _{ac}/2e \, (n=1,2,3,\ldots )$ and $\omega _{ac}=2 \pi f$. At a certain power level, steps of much lower amplitude were visible at $V_{n/2}=(n/2) (\hbar \omega _{ac}/2e)$, corresponding to subharmonics of the frequency of the microwave signal. Then, as it is expected for a standard JJ, the amplitude of the critical current and of the current steps oscillated when varying the irradiation power. The critical currents of the studied contacts were in the range ($0.44 \div 2.82)\, {\mathrm {mA}}$.

**Figure 3:**
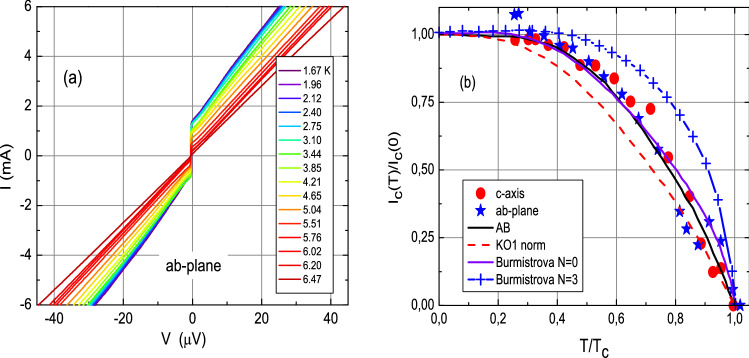
(**a**) $I-V$ characteristics of a ${\hbox {Pb}_{0.6}\hbox {In}_{0.4}}$/$\hbox {Ba}_{0.6}\hbox {K}_{0.4}\hbox {(FeAs)}_2$ point-contact Josephson junction, recorded in zero magnetic and zero microwave fields at different temperatures. (**b**) Symbols: measured normalized critical currents $I_c (T)$/$I_c (0)$ as a function of the normalized temperature *T*/$T_c$ for a PC with a current injection along the *ab* plane (blue stars) and for a contact with current along the *c*-axis (red circles). Lines: theoretical dependencies for (1) an Ambegaokar–Baratoff (AB)^36^ *S*/*I*/*S* tunnel contact between “ordinary” superconductors (black, solid), (2) contacts with current injection parallel to the *ab* plane between an “ordinary” *S* and a two-band $s \pm$ superconductor^15^ in the case of a direct microshort (solid line, violet) and a tunnel contact (line and crosses, blue) and (3) for Kulik–Omelyanchuk (KO1) dirty *S*/*S* microshorts^37^ (red dashed line).

We measured the temperature dependencies of the critical currents in the range $T = (1.7 \div 6.8) \, K$ for several Josephson PCs. The results are shown in Fig. 3. Figure 3a shows the $I-V$ characteristics of one of the PCs at different temperatures. It can be seen that these characteristics have a noticeable hysteresis ($+I_c \ne -I_c$) and differ from the hyperbolic-dependent characteristic of the resistively shunted model for a fixed current. The PC resistance practically did not change during the measurements. In Fig. 3b, the symbols show the measured dependencies of the normalized critical currents $I_c (T)$/$I_c (0)$ versus the normalized temperature *T*/$T_c$ for two contacts with currents flowing along the *ab* plane (blue stars, cooling) and along the *c* axis (red circles, heating).

**Figure 4:**
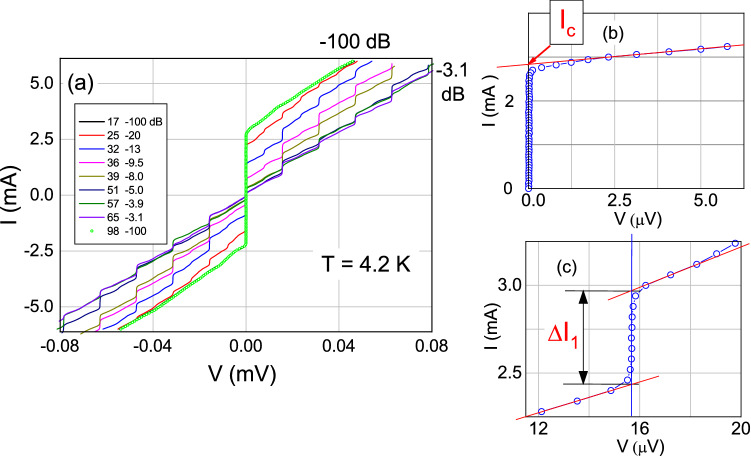
(**a**) Some examples of current-voltage characteristics of the ${\hbox {Pb}_{0.6}\hbox {In}_{0.4}}$/ $\hbox {Ba}_{0.6}\hbox {K}_{0.4}\hbox {(FeAs)}_2$Josephson point-contact No. 1 recorded at $f= 7.6 \, {\mathrm {GHz}}$ and different levels of microwave power. The legend reports the numbers of the $I-V$ characteristics and the relevant attenuation of the microwave power in decibels. Panels (**b**) and (**c**) show the method for measuring the critical current and the amplitude of the current steps. (**b**) Initial part of $I-V$ curve 17. The thin straight line shows the extrapolation of the initial part of the $I-V$ characteristic made for determining the critical current $I_c$ of the contact $I_c=2.82 \, {\mathrm {mA}}$. (**c**) Part of curve 32. The amplitude of the step was determined by the points of intersection of the vertical line passing through the center of the step with the lines extrapolating the $I-V$ curve.

**Figure 5:**
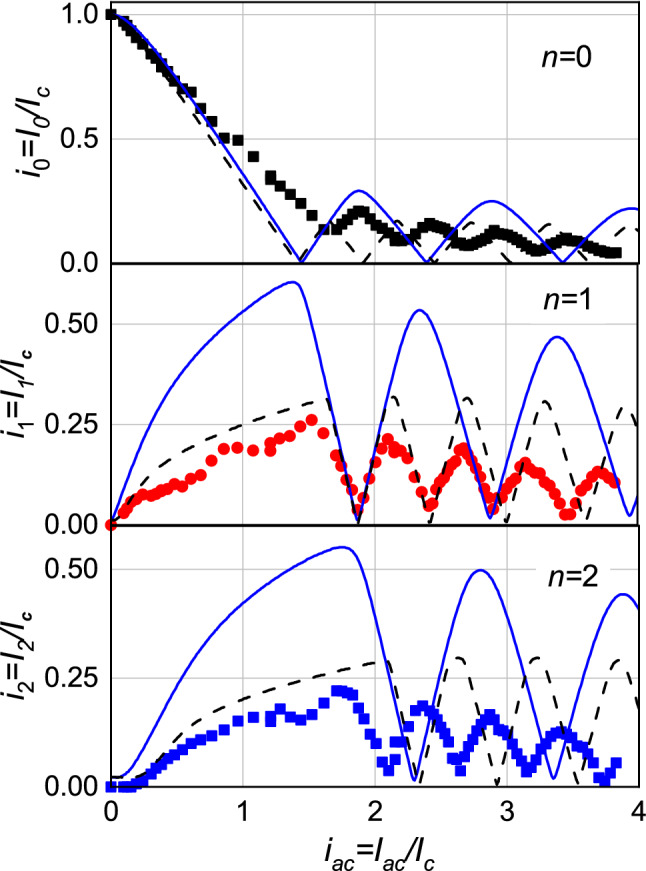
Symbols: experimental dependencies of the normalized critical current and amplitudes of the first current steps for PC No. 1, plotted as $i_n^{exp}(k \sqrt{P})=I_{n} (k \sqrt{P})/I_c (P = 0) \, (n=0,1,2)$. Lines: approximation of experimental dependencies obtained from the RSJ Eq. (1) with $\Omega _{VAC}=0.35$. The solid blue one has been obtained for the current-phase relationship $I_s=I_c \sin (\varphi )$, the dashed black one for $I_s=I_c \sin (2\varphi )$. The *x*-axis for the dependencies $i_n^{exp}(k \sqrt{P})$ and $i_n^{calc-2 \varphi }(hi_{ac})$ was rescaled so that the first minima of $i_1^{exp}(k \sqrt{P})$ and $i_1^{calc-2 \varphi }(h i_{ac})$ coincide with the first minimum of $i_1^{calc- \varphi }(i_{ac})$. The coefficients $k=3.83$ and $h=1.18$ were found for these two cases.

Figure 4a shows some typical current-voltage characteristics of a Josephson point-contact, labelled No. 1, between Pb$_{0.6}$In$_{0.4}$ and Ba$_{0.6}$K$_{0.4}$(FeAs)$_2$, recorded at increasing microwave power *P* from zero (CVC 17, attenuation: $100 \,$ dB) to a value that almost completely suppresses the critical current $I_c$ of the contact (CVC 65, attenuation: $3.1 \,$ dB). It can be seen that the branches of the $I-V$ curves at positive ($+V$) and negative ($-V$) voltages are symmetrical and almost linear at low bias. It is also worth noticing that some features of the curves are not described by the RSJ model reported in Eq. (1) as, for example, the shape of the CVC and the hysteresis of the curve 17 (exactly superimposed by curve 98), recorded at zero power, where $+I_c/-I_c \approx 1.23$ or the fact that the curves near $I \approx I_c$ are slightly rounded. At the same time, it is clearly seen that despite the difference in the shape of the $I-V$ characteristic from the hyperbolic one, which follows from the RSJ model^26^, we can also clearly observe the oscillations of the amplitude of the current steps corresponding to the harmonics and subharmonics of the MW signal when the power *P* is changed. A total of 81 $I-V$ curves were recorded for this PC at different powers, and the first (CVC 17, zero MW power), and last (CVC 98, zero MW power) curve are practically coincident, thus showing that the characteristics of the junction did not change during the measurement. Symbols in Fig. 4b and c show the low-voltage part of CVC 17 and part of CVC 32 in the vicinity of the fist current step, respectively. The thin red line in panel (**b**) indicates the extrapolation of the low-voltage part of the $I-V$ curve towards $V=0$, while in panel (**c**), the red lines indicate the extrapolation of the $I-V$ curve measured closely to the current step in the region of this step. The critical current $I_c$ and the amplitude of the step were determined by the intersection of these lines with the current axis and with a vertical line drawn along the center of the step, respectively, which is a standard method for determining the amplitudes of the current steps $I_n \, (n=0,1,2, \ldots )$^34^.

By analyzing in this way all the $I-V$ curves recorded at different power levels, it is possible to extract the dependence of the amplitude of the current steps $I_n (P)$ normalized to the critical current at zero MW power, $I_c (0)$, as a function of the square root of the irradiation power $\sqrt{P}$, that is: $i_n^{exp}=I_n^{exp}(\sqrt{P})/I_c (0)$. The $i_n^{exp}(k \sqrt{P})$ dependence of the first three current steps $(n=0,1,2)$ of the point contact No 1, typical of all the junctions we measured, is reported in Fig. 5 as symbols, where *k* is a scaling constant always necessary for this experimental geometry in order to account for the unknown coupling between the microwave and the junction. The proper choice of this scaling constant will be discussed later. We can see that in the PC No.1 the minimum values of the $i_n^{exp}(k \sqrt{P})$ are not zero. Usually, it is difficult to observe the zeroes in the amplitude oscillations of $i_n^{exp} (\sqrt{P})$ in the experiments, since the amplitude of the steps, which depends on *P*, changes very sharply near the zero values. Moreover, the reason for this deviation from the standard RSJ model may be the presence of higher harmonics $(n>1)$ in the superconducting current of the contact ($I_s \sim \sum _{n} I_{c,n} \sin (n \varphi )$) or, in the worst case, to a small ($\le 10 \%$ of $I_c$) superconducting, non-Josephson current which has not disappeared under microwave irradiation. For all the other junctions studied in this work, the superconducting, non-Josephson current was either absent or less than $5 \%$ of $I_c$, as it will be reported later in Table 1.

It is obvious that the suppression of $I_c$ and the current steps in the $I-V$ characteristic produced by the microwave signal are not associated with the penetration of Josephson vortices into the contact. This is indicated by the multiple oscillations of the current steps of $i_n^{exp} (\sqrt{P})$ in the microwave field. Indeed, the current steps arising from the motion of magnetic vortices in the contact do not oscillate with increasing *P*^35^.

## Discussion

Let us try to approximate the measured dependencies $I_c (T)$/$I_c (0)$ shown in Fig. 3b (symbols) by known models. In this figure, the lines show the calculated normalized dependencies according to: (1) Ambegaokar–Baratoff (AB)  theory^36^ for a tunnel contact between “ordinary” superconductors *S*/*I*/*S* (*I*-insulator layer) (black, solid) and (2) Burmistrova et al.^15^ model (Fig. 11 (c) of Ref.^15^) for a contact between an “ordinary” superconductor *S* and a two-band superconductor with $s\pm$ OP symmetry with a current injection parallel to the *ab* plane in the case of a microshort ($N = 0$ - where *N* is the number of insulating layers between the *S* and $s \pm$ superconductors) (solid line, violet), which are the model curves closest to the measured dependencies. For comparison we also show the Kulik–Omelyanchuk dependence for a “dirty” *S*/*S* microshort (KO1)^37^ (red, dashed) and the one by Burmistrova et al.^15^ for a tunnel $S/I/s \pm$ contact with *ab* current direction ($N = 3$) (blu line + symbol). It can be seen that the measured dependencies for an ordinary *s* symmetry of the OP in FeBS support a tunneling conductivity in our PC, and for a $s\pm$ OP symmetry they support the absence of a tunnel barrier in these contacts. At the same time, the measured dependencies differ significantly from similar dependencies for *S*/*S* microshorts in the “dirty” limit (KO1)^37^ and this is even more true for microshorts in the “clean” limit (KO2)^38^. Note that the values of the Josephson junction parameters for the dependencies calculated by Burmistrova et al. lead to the $\sin (\varphi )$ proportionality of the Josephson current both for the microshort and for the tunnel contact with $\varphi$= 0 or $\pi$ (Fig. 7 of Ref.^15^). Nevertheless, the shape of the low-voltage part of the $I-V$ characteristics of all measured PCs is more typical of microshorts than of tunnel contacts, in accordance to what is generally expected for point contacts, that are usually modeled as weak links. For a $S/s \pm$ contact perpendicular to the *ab* plane ($c-$ or *z*-axis contact), $I_c$ is always proportional to $\sin (\varphi )$. The critical current of the tunnel contacts $S/I/s \pm$ flowing both in the *ab* plane and along the *c* axis decreases with temperature more smoothly than what expected from AB theory for the *S*/*I*/*S* tunnel contact. Thus, the results of the measurements of $I_c (T)$ seem to be compatible with a $\sin \varphi$ dependence of the superconducting Josephson current in the Pb$_{0.6}$In$_{0.4}$/Ba$_{0.6}$K$_{0.4}$ (FeAs)$_2$ PCs studied by us, regardless of the direction and type of contact. It is worth noticing here that interesting deviations from the Ambegaokar–Baratoff behavior are predicted in the case of junctions between two identical FeBS^39^. However, (1) our junctions are of the $S/s \pm$ type and not of $s \pm /s \pm$ one and (2) the apparent similarity with the AB behavior shown in Fig. 3b should be considered accidental because, as already mentioned, the point-contact nature of the junction and the low voltage part of the $I-V$ characteristic, indicate a microshort nature of our contacts, that is quite well represented by the curve by Burmistrova et al.^15^ for the case $N=0$.

To really distinguish whether the superconducting Josephson current $I_s$ is proportional to $\sin \varphi$ or $\sin (2 \varphi )$, it is necessary to measure several oscillations of the amplitude of the first three current steps $i_n^{exp}(\sqrt{P})$ $n=0,1,2$^15,25^ in the current–voltage characteristics, in order to find the coefficient *k* that accounts for the microwave power loss, and compare the oscillation periods of the $i_n^{exp}(k \sqrt{P})$ thus obtained, with those of the calculated dependencies $i_n^{calc}(i_{ac})$, for both current-phase relationships $I_s \propto \sin (\varphi )$ and $I_s \propto \sin (2 \varphi )$. For calculating the corresponding dependencies, we solved the differential equation of the RSJ model without the terms taking into account the effect of noise, capacitance and inductance^26^. Within this approximation, the contact is represented as an ideal Josephson junction, through which only the supercurrent $I_s=I_c \sin (d\varphi )$ ($d=1, 2$) flows, and the ohmic resistance, $R_N$ is connected in parallel to the JJ. The calculation of the contact $I-V$ characteristic is reduced to solving a relatively simple differential equation^26,40^:

1 $$\begin{aligned} \frac{d \varphi }{d \tau }= & {} i+i_{ac} \sin \Omega \tau - \sin (d \varphi ),\ d=1,2 \nonumber \\ \tau= & {} \left(\frac{2e}{\hbar }I_c R_N\right)\,t \qquad \Omega =2 \pi f /\left(\frac{2e}{\hbar }I_c R_N\right) \end{aligned}$$

where *i* is the *dc*-current normalized to $I_c$, $\varphi$ is the phase difference of the order parameter in the Josephson junction electrodes, $\tau$ is the normalized time. When solving this equation, the constant *i* and alternating $i_{ac}$ currents were set, $\varphi (\tau )$ was found, and the constant voltage $\nu$, normalized to $I_c R_N$ was equal to the time-averaged oscillations $\langle d \varphi / d \tau \rangle$. The result is a normalized $I-V$ characteristic $\nu (i)$. Further processing of the calculated current-voltage characteristics allowed us to obtain the desired normalized current dependencies, $i_n^{calc}(i_{ac})$, $(n=0,1,2)$. The calculated dependencies were compared with the corresponding measured dependencies $i_n^{exp}(k \sqrt{P})$ by selecting the coefficient *k*, which multiplies $\sqrt{P}$, so that the first minima of the calculated and measured oscillations of the first current step, $i_1$ coincide. The coefficient *k* takes into account the power loss of the microwave signal in the cable and in the path between the antenna and the point contact. This procedure made it possible to find the $i_{ac}$ current induced in the point contact. In order to calculate $i_n^{calc}(i_{ac})$ from the RSJ Eq. (1), it is necessary to know the normalized frequency of the microwave radiation, $\Omega$ whose value is determined by the characteristic Josephson junction voltage $V_c=I_c R_N$, the only characteristic parameter of the junction that has to be found in order to fit the period of the measured dependence $i_n^{exp}(k \sqrt{P})$. Usually, $V_c$ is determined from the junction current–voltage characteristic by assuming that $I_c$ is not affected by noise, while $R_N$ can be obtained either by suppressing the superconductivity in the contact by applying a magnetic field, or by looking at the linear part of the $I-V$ curve at voltages of the order of the sum of the energy gaps of the contact electrodes. For our type of junctions, $V \propto 2(\Delta _{\mathrm{PbIn}}+\Delta _{\mathrm{BaKFeAs}})/e \approx 20 \, {\mathrm {mV}}$, where $\Delta _{\mathrm{PbIn}}$ and $\Delta _{\mathrm{BaKFeAs}}$ are the energy gaps of the two electrodes. Here, none of these methods is viable due to the high critical magnetic field of Ba$_{0.6}$K$_{0.4}$(FeAs)$_2$, which exceeds $30 \, {\mathrm {T}}$^41^ in the first case, and because at $V > 100$μV, the $I-V$ curves start changing because of heating, in the second. Thus, as it has also been done in the works published previously^15,16–25^, as a first approximation, the $I_c$ and $R_N$ of the studied point contacts were determined only from the initial part of the current–voltage characteristic. For example, from the right branch of the CVC 17 of the junction No. 1 it follows that $I_c=2.82 \, {\mathrm {mA}}$, $R_N=0.016 \, \Omega$ and $V_c=45.1$ μV. It is worth noticing that the value obtained in this way for $V_c$ differs by more than two orders of magnitude from the corresponding characteristics of the Josephson junctions in ordinary superconductors^26^. The value of the normalized frequency of electromagnetic radiation reported in Eq. (1), and obtained from the low-voltage portion of the $I-V$ curve is denoted by $\Omega _{VAC}=V_{n=1}/V_c=0.35$, where $V_{n=1}$ is the voltage at which the first current step occurs. For Josephson junctions between ordinary superconductors that feature a current–voltage characteristic of hyperbolic shape, $V_c$ and its corresponding $\Omega$ are ideally determined by the energy gaps of the electrodes. Since in our case, $\Delta _{\mathrm{PbIn}}<<\Delta _{\mathrm{BaKFeAs}}$, the value of $V_{c0}$ is approximately equal to^42^:

2 $$\begin{aligned} V_{c0}\approx \frac{\Delta _{\mathrm{PbIn}}}{e}ln \frac{4 \Delta _{\mathrm{BaKFeAs}}}{\Delta _{\mathrm{PbIn}}} \end{aligned}$$

The energy gaps of ${\hbox {Pb}_{0.6}\hbox {In}_{0.4}}$and $\hbox {Ba}_{0.6}\hbox {K}_{0.4}\hbox {(FeAs)}_2$are $\Delta _{\mathrm{PbIn}}= 0.62 \, {\mathrm {meV}}$^43^ and $\Delta _{\mathrm{BaKFeAs}}= 6$ and $12 \, {\mathrm {meV}}$, respectively^44,45^. In a point contact between these two superconductors, the value $V_{c0} \approx (2.3-2.7) \, {\mathrm {mV}}$, tentatively obtained from Eq. (2), exceeds the characteristic voltage of the real junction by more than two orders of magnitude. This can be explained by the fact that, in case of $s \pm$ symmetry of the order parameter in the FeBS electrode, the $V_c$ value of the contact can significantly differ from $V_{c0}$, because the interference between the supercurrents flowing in the Fermi pockets with opposite sign of the order parameter reduces the total Josephson current of the point contact, $I_s=I_{s+}-I_{s-}$. Therefore, to obtain a noticeable $I_c$ in the contact, it is necessary to reduce its resistance $R_N$ and, as a consequence, this leads to low values of $V_c$. It is interesting to note that $V_c$ of the order of several tens of $\mu V$ are typical in Josephson junctions between ordinary superconductors and those with unusual pairing like cuprates^42^, and FeBS^15,16–25^. On the other hand, however, small values of $V_c$ are not a clear evidence for the $s \pm$ symmetry of the order parameter in $\hbox {Ba}_{0.6}\hbox {K}_{0.4}\hbox {(FeAs)}_2$, because similar values have also been observed in Josephson junctions between “ordinary” superconductors^46^.

According to the considerations and estimates reported above, using the $V_c$ value obtained from the low-voltage part of the $I-V$ curve to find the parameter $\Omega$ necessary for calculating $i_n^{calc}(i_{ac})$, appears somehow incorrect. At the same time, however, the oscillations of the Josephson current as a function of the microwave power $i_n^{exp}(\sqrt{P})$ in the case of S/FeBS junctions can be rather accurately approximated by the dependencies calculated from Eq. (1), at the corresponding choice of the normalized frequency $\Omega$^25^. Therefore, let us try, also in this case, to describe the oscillations of the current step of the point contact No. 1 by using $\Omega _{VAC}=0.35$, as determined before. We calculated, from Eq. (1) and with this value of $\Omega _{VAC}$, the dependence $i_n^{calc}(i_{ac})$ for both $I_s=I_c\sin (\varphi )$ and $I_s=I_c\sin (2 \varphi )$, that we call $i_n^{calc- \varphi }(i_{ac})$ and $i_n^{calc- 2 \varphi }(i_{ac})$, respectively. In Fig. 5 by symbols we show the amplitude dependencies of the first three current steps as a function of $\sqrt{P}$, $i_n^{exp}(k \sqrt{P})$ $(n=0,1,2)$ and by lines the calculated $i_n^{calc- \varphi }(i_{ac})$ (solid blue) and $i_n^{calc- 2 \varphi }(i_{ac})$ (dashed black). The axis *x* for the dependencies $i_n^{exp}(k \sqrt{P})$ and $i_n^{calc-2 \varphi }(i_{ac})$ was rescaled so that the first minimum of the curves $i_1^{exp}(k \sqrt{P})$ and $i_1^{calc-2 \varphi }(i_{ac})$ coincides with the first minimum of $i_1^{calc- \varphi }(i_{ac})$. This was achieved by multiplying the *x* axis of the measured $i_n^{exp}(k \sqrt{P})$ by the coefficient $k=3.83$. On the other hand, in the case of $i_n^{calc- 2 \varphi }(i_{ac})$, the *x* axis was multiplied by $h=1.18$. It can be seen that for the first current step ($n=1$), the period of the calculated dependence $i_1^{calc- 2 \varphi }(h i_{ac})$ is close to the period of $i_1^{exp}(k \sqrt{P})$. However, for $n=0$ and 2, the periods and/or the positions of minima of these dependencies differ noticeably from the calculated ones. As for the periods of the calculated $i_n^{calc-\varphi }(i_{ac})$, the difference from the measured ones is great, for all the three steps. Thus, it is clear that in this case the power dependencies of the current steps amplitude, calculated by using $\Omega _{VAC}$, are not able to describe the experimental results $i_n^{exp}(k \sqrt{P}), \, n=0,1,2$. Let us now consider the amplitude of the oscillations: it is evident that the calculated amplitudes are much higher than the experimental ones, and this is due to the fact that, as it is well known, the amplitudes of the oscillations depend on the level of the noise signal in the contact, which, however, does not affect their period^26,34,47^. Since we are here interested only in the oscillation period, because it is this characteristic that is directly connected to the phase dependence of the Josephson superconducting current, for the calculations of $i_n^{calc}(i_{ac})$ we solved Eq. (1) without considering the noise current.

It is now worth noticing, after what has just been shown above, that there is a number of studies of Josephson contacts between “ordinary” and high-temperature superconductors based on Cu or Fe, where it was also not possible to approximate the oscillations of the current steps as a function of applied microwave power by using the renormalized frequency $\Omega$ obtained from the current-voltage characteristics. In those cases, in order to reproduce the experimental behavior $i_n^{exp}(\sqrt{P})$, the authors handpicked the $\Omega$ value and the contact capacity^23,48^. As it has already been mentioned before, in our case the problems arising in the correct determination of $\Omega =V_{n=1}/V_{c}$, necessary for approximating the measured dependencies $i_n^{exp}(\sqrt{P})$ by means of the RSJ model, are associated to a number of deviations of the investigated Josephson point contacts from the ideal behavior and with the determination of the $V_c=I_c R_N$ value from the low-voltage part of the $I-V$ curve. In particular they are: (1) the presence of noise and the consequent errors in the determination of the critical current value $I_c$, (2) the impossibility to have access to the actual value of the contact resistance $R_N$ because of the experimental limitations already discussed before, and (3) the effect of the contact capacitance which affects the oscillation period of the current steps. The value of the capacity of our point contacts, however, cannot be determined because, for example, the size and characteristics of the transition layer cannot be estimated. At the same time, almost all the current–voltage characteristics we measured showed a considerable hysteresis, which can be ascribed to the presence of a contact capacitance.

**Figure 6:**
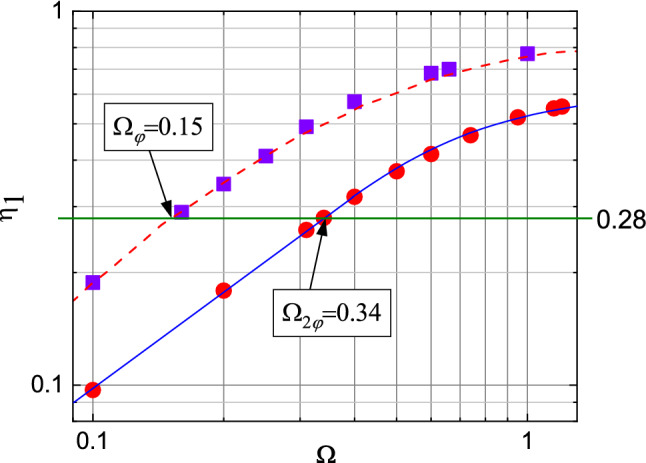
Dependencies of the normalized oscillation period of the first current step $\eta _1(\Omega )=(i_1^{(2)}-i_1^{(1)})/i_1^{(1)}$ on the $I-V$ characteristic as a function of the normalized frequency, $\Omega$. $i_1^{(1)}$ and $i_1^{(2)}$ are the first and second minimum in the amplitude dependence of the first current step, calculated from Eq. (1). Red dashed line: $\eta _1(\Omega )$ dependence calculated by Likharev^49^ for $I_s=I_c\sin (\varphi )$. Violet squares and red circles: $\eta _1(\Omega )$ for $I_s=I_c\sin (\varphi )$ and $I_s=I_c\sin (2 \varphi )$ calculated in this work, respectively. Solid blue line: approximations of the values indicated by the red circles. For point contact No. 1 we obtained $\eta _1=0.28$, which corresponds to $\Omega _{\varphi }=0.15$ and $\Omega _{2 \varphi }=0.34$.

**Figure 7:**
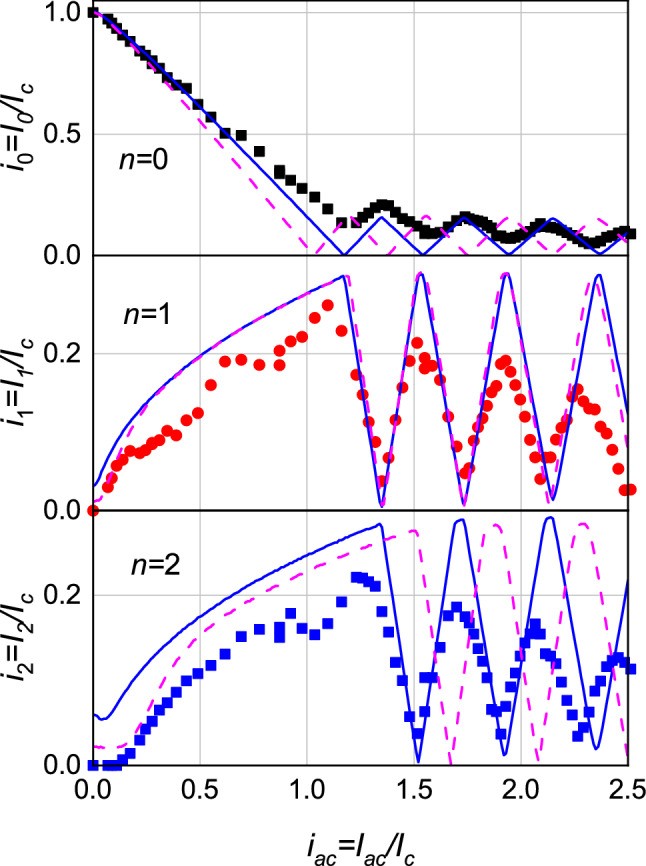
Fit of the measured oscillations of the current steps of point contact No. 1 (symbols) by means of the dependencies calculated from the RSJ model, reported in Eq. (1), by using $\Omega _\varphi =0.15$ ($I_s=I_c\sin (\varphi )$, solid blue line) and $\Omega _{2 \varphi }=0.34$ ($I_s=I_c\sin (2 \varphi )$, dash magenta line). $\Omega _\varphi$ and $\Omega _{2 \varphi }$ were determined from the graph of Fig. 6. The abscissae of graphs $i_n^{exp}(k^* \sqrt{P})$ and $i_n^{calc- 2\varphi }(h^* i_{ac})$ were scaled by the coefficients $k^*=2.76$ and $h^*=1.15$, respectively, to have the first minimum of all dependencies $i_1$ coincident.

The three issues reported above affect the value of $\Omega$ and, consequently, the period of the calculated dependencies $i_n^{calc}(i_{ac})$. There is a way, however, to bypass these problems and reliably determine whether the current-phase relationship is of the type $I_s=I_c\sin (\varphi )$ or $I_s=I_c\sin (2 \varphi )$: $\Omega$ must be *directly* determined from the period of the measured oscillations of the current steps, $i_n^{exp}(\sqrt{P})$, thus automatically accounting for problems (1), (2) and (3). For accomplishing this task, we consider the work done by Likharev and Semenov and reported in Ref.^49^. There, the authors started from the RSJ model reported in Eq. (1) with a current-phase relationship of the type $I_s=I_c\sin (\varphi )$, and calculated, as a function of $\Omega$, the dependence of the normalized oscillation periods for several current steps of the current-voltage characteristics, $\eta _n=(i_n^{(2)}-i_n^{(1)})/i_n^{(1)}$ where $i_n^{(1)}$ and $i_n^{(2)}$ are the first and second minimum in the power dependence of the $n-th$ current step $i_n(i_{ac})$, respectively. From the experimental power dependence of the first step, $i_1^{exp}(\sqrt{P})$, reported in Fig. 5, we obtained the value $\eta _1^{exp}=0.28$. From the graph reported in Ref.^49^, part of which is reproduced in Fig. 6 (squares and dashed line), we were able to extract the normalized frequency of the microwave radiation as $\Omega _\varphi \approx 0.15$. We then calculated the power dependencies $i_n^{calc- \varphi }(i_{ac})$ $(n=0,1,2)$ from Eq. (1) by using the $\Omega _\varphi$ just found and $I_s=I_c\sin (\varphi )$. The obtained result, after using $k^*=2.76$ as the rescaling coefficient for $\sqrt{P}$, is reported in Fig. 7 (blue line). It is possible to notice that, in contrast to what we have shown in Fig. 5, both the periods and the minima of the measured oscillations, $i_n^{exp}(k \sqrt{P})$ (symbols) coincide for all the three steps with those of the calculated dependencies, $i_n^{calc- \varphi }(i_{ac})$, reported as blue lines, which proves that the Josephson current-phase relationship in point contact No. 1 is of the type $I_s=I_c\sin (\varphi )$.

Let us also try, however, to approximate the dependencies measured for point contact No. 1, $i_n^{exp}(\sqrt{P})$, by using the RSJ model with $I_s=I_c\sin (2 \varphi )$. In this case, it is first necessary to determine the corresponding value $\Omega _{2 \varphi }$. We thus calculated the current-voltage characteristics from Eq. (1) with $I_s=I_c\sin (2 \varphi )$ $(i_{ac}=0 \div 8)$ for several values of $\Omega$ and obtained the dependence of the normalized oscillation period of the first step as a function of $\Omega$, $\eta _1^*(\Omega )$, shown in Fig. 6 (circles and corresponding line). For the $i_n^{exp}(k^* \sqrt{P})$ shown in Fig. 7, with $\eta _1^{exp}=0.28$ we obtained $\Omega _{2 \varphi }=0.34$ (Fig. 6). As it has been done before, the calculated dependencies $i_n^{calc- 2 \varphi }(h^* i_{ac})$ were adjusted to the measured ones (symbols) by letting coincide the first minima of the first current steps $i_1$ for all the three dependencies $i_1^{exp}$, $i_1^{calc-\varphi }$ and $i_1^{calc-2\varphi }$, with a fitting coefficient $h^*=1.15$, as shown in Fig. 7 by magenta dashed lines. The oscillation period $i_1^{exp}(h^* \sqrt{P})$ is reproduced very well but it is clear that the relation $I_s=I_c\sin (2 \varphi )$ cannot adequately reproduce the experimental $i_n^{exp}(\sqrt{P})$ curves for all the three steps measured in our experiment.

**Table 1:** Summary of the main characteristics of the point-contact Josephson junctions studied in this work. $I_c$ is the critical current of the contact, $R_N$ its resistance, determined from the low-voltage part of $I-V$ curve, $V_c=I_c R_N$ is the characteristic voltage of the junction, $I_{non-J}/I_c$ is the percentage of the superconducting non-Josephson current, $\Omega _{VAC}$ the normalized frequency of the microwave radiation as determined directly from the current-voltage characteristic. $\eta _1$, $\Omega _\varphi$ and $\Omega _{2 \varphi }$, which are shown in Fig. 6, are the normalized oscillation period of the first current step and the normalized frequencies of the microwave radiation, found from the oscillation periods of the first current steps for the two different $I_s(\varphi )$ relationships, respectively. For contact No. 8 there is no intersection between $\eta _1$ and $\Omega _{2 \phi }$.

No.	$+I_c$ (μA)	$+I_c/-I_c$	$R_N \, ({ {\Omega })}$	$+V_c \,$ (μV)	$I_{non-J}/I_c$	$\Omega _{VAC}$	$\eta _1$	$\Omega _\varphi$	$\Omega _{2 \varphi }$	$f \, ({\mathrm {GHz}})$	Notes
1	2820	1.23	0.016	45.1	$\le 10 \%$	0.35	0.28	0.15	0.34	7.6	$\Omega _{VAC}\approx \Omega _{2 \varphi }$
2	1830	1.095	0.0057	10.43	$\le 7 \%$	1.32	0.42	0.25	0.6	6.68	*c*-axis
3	1390	1.97	0.0055	7.65	$\le 5 \%$	0.51	0.49	0.33	0.84	1.91	
4	1270	1.02	0.014	17.78	$\le 3 \%$	0.60	0.48	0.31	0.8	5.15	
5	1050	0.88	0.046	48.3	0	0.33	0.36	0.22	0.5	7.6	
6	658	1.39	0.1	65.8	$\le 5 \%$	0.24	0.41	0.25	0.6	7.6	$\Omega _{VAC}\approx \Omega _{\varphi }$
7	508	1.03	0.33	167.6	0	0.09	0.29	0.16	0.34	7.6	
8	440	0.97	0.08	35.2	$\le 3 \%$	0.45	0.68	0.6	-	7.6	

Analogous conclusions and similar results were obtained by studying several other ${\hbox {Pb}_{0.6}\hbox {In}_{0.4}}$/$\hbox {Ba}_{0.6}\hbox {K}_{0.4}\hbox {(FeAs)}_2$point contact Josephson junctions. Table 1 shows the characteristic parameters obtained from the point contacts studied in this paper while the $I-V$ characteristics and the behavior of the current steps as a function of microwave power for contacts No. 2 and 4 are reported in the Supplementary Information. The critical current $I_c$ of the contacts was in the range $I_c \approx (0.44-2.82) \, {\mathrm {mA}}$ and the resistance $R_N\approx (5.5 \div 330)$ mΩ. All the $I-V$ characteristics could not be adequately described by the RSJ model, as discussed for point contact No. 1. Let us note that, even though for point contact No. 1, $\Omega _{VAC}\approx \Omega _{2 \varphi }$ and for No. 6, $\Omega _{VAC}\approx \Omega _{\varphi }$ for the first step, the periods of the experimental dependencies $i_n^{exp}(\sqrt{P})$ with $(n=0,1,2)$ of *all* the junctions could be fitted by the calculated $i_n^{calc- \varphi }(h i_{ac})$ dependencies only by using the $\Omega _{\varphi }$ found from the oscillation period of the first current step. Therefore, the coincidence of $\Omega _{VAC}$ with $\Omega _{\varphi }$ or $\Omega _{2 \varphi }$ has to be considered accidental. The present results show that the superconducting currents of the investigated ${\hbox {Pb}_{0.6}\hbox {In}_{0.4}}$/$\hbox {Ba}_{0.6}\hbox {K}_{0.4}\hbox {(FeAs)}_2$ Josephson junctions are proportional to the sine of the phase difference of the order parameter in the electrodes, $I_s=I_c\sin (\varphi )$.

## Conclusions

In conclusion, stable point-contact Josephson junctions were obtained between an “ordinary” superconductor *S* ($S=$ ${\hbox {Pb}_{0.6}\hbox {In}_{0.4}}$with critical temperature $T_c \approx 6.6 \, {\mathrm {K}}$) and the edge (*ab*-plane contacts) or face (*c*-axis contacts) of a single crystal of the optimally doped Fe-based superconductor $\hbox {Ba}_{0.6}\hbox {K}_{0.4}\hbox {(FeAs)}_2$with $T_c \approx 38.5 \, {\mathrm {K}}$. The current-voltage characteristics, critical currents $I_c$, contact resistances $R_N$, dependencies of the point contact critical currents on temperature $I_c (T)$, and amplitude of the first current steps as a function of applied microwave power were thoroughly investigated at temperatures $T=(1.7 \div 6.8)\, {\mathrm {K}}$ and microwave frequencies in the range $f \approx (1.5 \div 8) \, {\mathrm {GHz}}$. It was found that the temperature dependencies of the critical current $I_c (T)$ for current flowing along the *ab* plane and along the *c* direction are close to the well-known Ambegaokar–Baratoff dependence for the *S*/*I*/*S* tunnel contact^36^(*I* is the insulator layer) and to the dependence calculated by Burmistrova et al.^15^ for microshorts $S/s \pm$ ($s \pm$ is a two-band superconductor with $s \pm$-wave symmetry of the order parameter) at certain values of the transparency of boundaries and thickness of the transition layer^15^. We showed that the main experimentally-derived characteristics of the point contacts estimated in the low voltage range of the current-voltage characteristics ($I_c$, $R_N$, and $V_c$) are not able to properly describe the oscillations of the current steps in the microwave field, when they are used for calculating these oscillations within the resistively shunted junction model both using the $I_c \sin (\varphi )$ and the $I_c \sin (2\varphi )$ current-phase relationships for the Josephson supercurrent. By revisiting an old procedure^49^, a new method has been proposed for determining the value of the normalized frequency of the microwave radiation, $\Omega =2 \pi f / (2eV_c / \hbar )$ that allows reproducing the behavior of the current steps as a function of the microwave power for current-phase relationships of the Josephson current of the type $I_s=I_c\sin (d \varphi )$, $d=1,2$. It is well known that the determination of the $I_s (\varphi )$ function can be very important in unraveling the symmetry of the order parameter and pairing interaction, especially in the case of the $s\pm$-wave symmetry. Thus, the method proposed here is capable of unambiguously determining the current-phase relationship, $I_s(\varphi )$ in Josephson contacts with Fe-based superconductors, bypassing the experimental limitations in the determination of $\Omega$ typical of these materials, and can be effectively employed to study, for example, the evolution of $I_s(\varphi )$ as a function of doping or chemical substitutions, in order to identify the conditions in which the symmetry of the order parameter can be more conveniently unveiled.

Not only, the method is more general since it can be applied for any type of current-phase relationship that can be introduced in Eq. (1) for calculating the normalized oscillation period of the first current step $\eta _1$ as a function of the normalized frequency $\Omega$, similarly to what is shown in Fig. 6, and thus could be extended also to other non conventional superconductors.

In the particular case of the optimally doped Ba$_{0.6}$K$_{0.4}$(FeAs)$_2$ presented here, our analysis allowed to conclude that the Josephson supercurrent of the investigated junctions is proportional to the $\mathrm{sine}$ of the phase difference of the order parameters in the electrodes, $I_s = I_c \sin (\varphi )$. Finally, by noticing that the low-voltage shape of all the investigated current–voltage curves is more typical of microshorts than tunnel contacts (as it is indeed expected for junctions of the point-contact type), we can say that, according to Ref.^15^, the behavior of the critical current as a function of temperature is compatible with a $s \pm$ symmetry of the order parameter. Moreover, the same range of hopping parameters that give rise to the temperature dependence of the critical current $I_c(T)$ that we reported experimentally, corresponds to a dominant $\sin (\varphi )$-dependence for the current-phase relationship in the case of $s \pm$ symmetry of the order parameter^15^, in agreement with our results.

## Methods

### Crystals

High-quality homogeneous, single-phase single crystals of Ba$_{0.6}$K$_{0.4}$(FeAs)$_2$ were grown from pure elements of Ba, K, Fe, As using FeAs flux and a cold finger positioned above the molten surface. Details are described in Ref.^29^. Separate single-crystal plates with a mirror-like surface, up to $2 \times 2 \,$ mm in size and up to several tens of microns thick, were split off from the grown ingot. The quality of single crystals was assessed using electron microscopy, X-ray diffraction studies, measurements of the composition, as well as measurements of the dependence of resistance and magnetic susceptibility on temperature. The *c* axis of the crystals was always perpendicular to the surface of the plates. For our research, we selected a homogeneous single-phase single crystal with a minimum width of the transition to the superconducting state. The dependencies of the resistance and magnetic susceptibility of this crystal are shown in Fig. 2. The critical temperature of the crystal is $T_c = 38.5 \, {\mathrm {K}}$, the width of the transition to the superconducting state (on the magnetic susceptibility curve) is  $\Delta T_c (10-90\%) = 0.3 \, {\mathrm {K}}$. The ratio of the resistance at room temperature and $T = 40 \, {\mathrm {K}}$ is $R(300\, {\mathrm {K}})$/$R(40\, {\mathrm {K}})=12$, the resistivity of the crystal $\rho (39\, \mathrm{K})\approx 40\,$ μΩcm. The quality of this crystal corresponds to the best known samples^27–33^. All measurements presented in this work were made on this crystal. We chose the Pb$_{1-y}$In$_y$ polycrystalline alloy (*y* = 0.4, $T_c \approx 6.6 \, K)$ as the “ordinary” single-band superconductor (*S*) for the second electrode of the Josephson junction. It allowed the reproducible production of stable Josephson contacts with Ba$_{1-x}$K$_x$(FeAs)$_2$ crystals^15,24^. This alloy was made from pieces of pure $(\ge 99.999)$ metals Pb and In, taken in the required proportion, according to the technology described in^43^. The characteristics of the obtained alloy are close to those reported in that work.

### Point contacts

The supercurrent in the Josephson junctions between Pb$_{0.6}$In$_{0.4}$ and a Ba$_{0.6}$K$_{0.4}$(FeAs)$_2$ single crystal was studied in point contacts tuned at $T = (1.8 \div 4.2) \, {\mathrm {K}}$. For preparing the PCs, a single-crystal FeBS plate was clamped by In on a foiled glass-fiber plastic substrate. The movable *S* electrode was soldered to a copper wire (Fig. 1). For a PC with a current along the *ab* plane, the FeBS plate was mounted so that the edge of the crystal was perpendicular to the plane of the substrate (Fig. 1a). For the *c*-axis contacts, the crystal was rotated by $90^\circ$ so that the PbIn-electrode was perpendicular to the *ab*-surface of the crystal (Fig. 1b). The PbIn-electrode of the PC in the form of a knife $\approx 1 \,$mm long or a pyramid was cut by a razor from a rectangular plate of Pb$_{1-y}$In$_y$^41^. A “fresh” cut of the working surface of PbIn-electrode was always used to create the PC. The surface of the FeBS crystal was always cleaved by cracking before the experiments. The electrodes, prepared in this way, were placed in a cryogenic insert equipped with a differential screw and a gearbox as shown in Fig. 1. The accuracy of the electrode movement was about 1 μm. With such a configuration of the electrodes, the current through the PC always flowed in the desired direction relative to the axes of the FeBS single crystal. The device prepared for measurements was placed in a cryostat. The Josephson junction was tuned after cooling to $T = 4.2 \, {\mathrm {K}}$ by moving the *S*-electrode. The Josephson nature of the pair current was verified by the presence of current steps in the $I-V$ characteristic of the contact when it was irradiated by a microwave signal.

### Current–voltage characteristics

The current–voltage characteristics of the point contacts were recorded using the standard four-probe scheme with the help of a Keithley 6221 current source and a Keithley 2182 nanovoltmeter. The sample temperature was continuously measured by a RuO$_2$ thermometer. An Agilent E8257D MW generator was used as the microwave radiation source. The radiation was supplied to the PC via a coax cable. The microwave signal was matched with the junction using an antenna at the end of the cable. After adjusting and mechanically stabilizing the PC, the current–voltage characteristics were recorded at: (1) increasing power *P* of the microwave signal and (2) varying temperature from the minimum to slightly above the critical temperature of Pb$_{0.6}$In$_{0.4}$. This made it possible to acquire the dependencies of the current steps on power $I_n (P)$ and of the critical current of the PC on temperature $I_c (T)$.

### Calculation of the microwave power dependencies of the amplitudes of the current steps

To calculate $i_n^{calc-d \varphi }(i_{ac})$ ($n = 0,1,2; \, d=1,2$), we used the differential Eq. (1) of the RSJ model of the Josephson contact without the terms taking into account the influence of noise, capacitance and inductance^26,40^. The solution of this equation by the Runge–Kutta method was carried out on a multiprocessor cluster. The program was written in Python^50^. The solution of this equation at different values of $i_{ac}$, $\Omega$ and *d* made it possible to obtain the $I-V$ characteristic of the contacts at the corresponding powers of the microwave signal $\sqrt{P} \propto i_{ac}$. Further processing of the current steps on the calculated $I-V$ characteristics using a MATLAB program allowed us to quickly obtain the desired normalized current dependencies, $i_n^{calc-d \varphi }(i_{ac})$, ($n = 0,1,2$).

## Supplementary Information

Supplementary Information.
