# Supplementary material for: Probing the current-phase relation in Josephson point-contact junctions between Pb0.6In0.4 and Ba0.6K0.4(FeAs)2 superconductors
Source: Sci Rep. 2021 Dec 14;11:23986. doi: 10.1038/s41598-021-00762-0 (PMC8671446; doi:10.1038/s41598-021-00762-0)
Supplement: Supplementary file 1 — Supplementary Information. [file 41598_2021_762_MOESM1_ESM.pdf]

SUPPLEMENTARY INFORMATION

Probing the current-phase relation in Josephson point-contact junctions between $Pb_{0.6}In_{0.4}$ and $Ba_{0.6}K_{0.4}(FeAs)_2$ superconductors

Valeriy A. Stepanov[1], **Chengtian Lin**[2†], **Renato S. Gonnelli**[3], **and Mauro Tortello**[3*]

[1]P.N. Lebedev Physical Institute of the Russian Academy of Sciences, Moscow, 119333, Russia

[2]Max-Planck-Institut für Festkörperforschung, D-70569 Stuttgart, Germany

[3]Dipartimento di Scienza Applicata e Tecnologia, Politecnico di Torino, Torino, 10129, Italy

[†]ct.lin@fkf.mpg.de   *mauro.tortello@polito.it

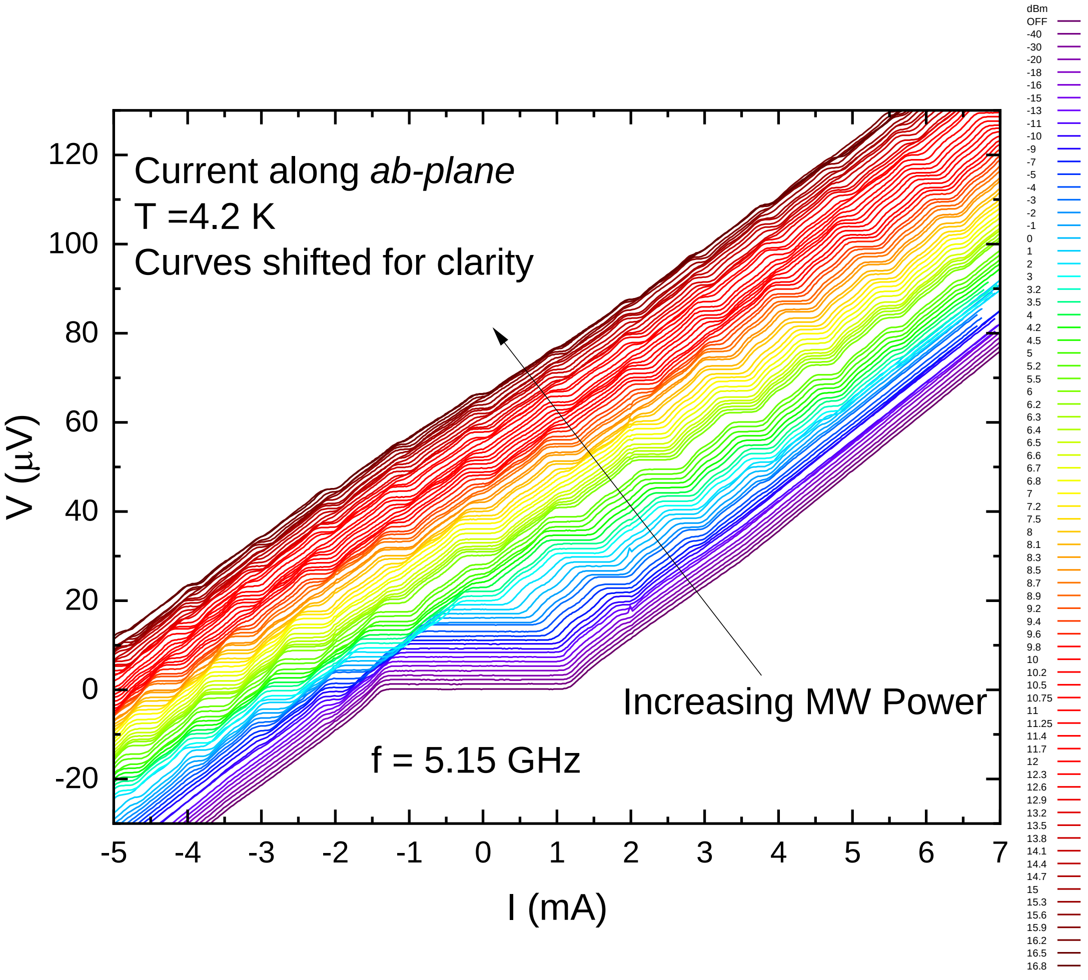

Supplementary Figure 1. Current-voltage characteristics of the $Pb_{0.6}In_{0.4}$ / $Ba_{0.6}K_{0.4}(FeAs)_2$ Josephson point-contact No. 4 recorded at *f* = 5.15 GHz and different levels of microwave power. The legend reports the attenuation of the microwave power in decibels.

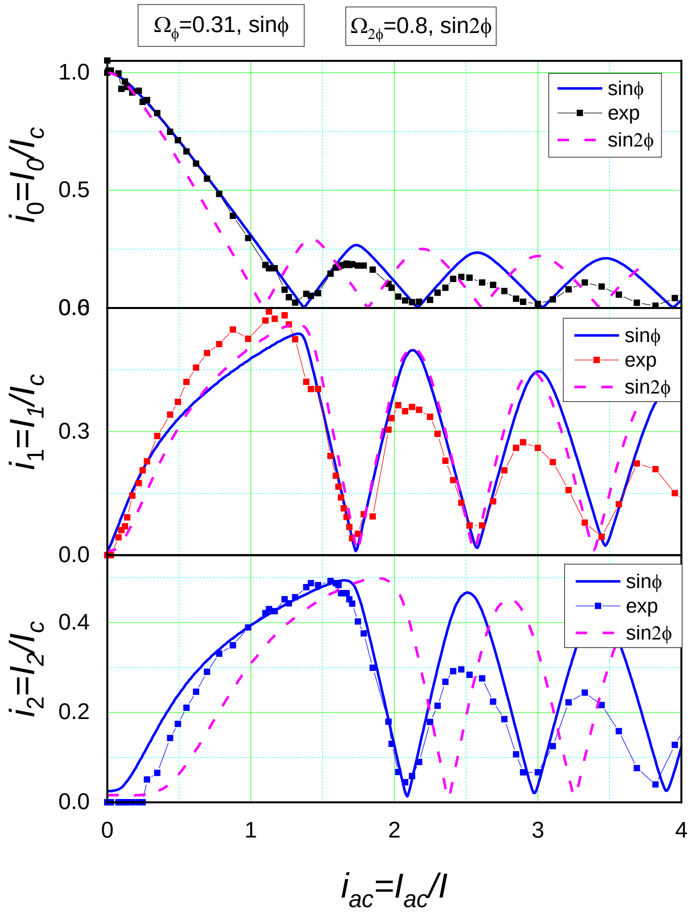

Supplementary Figure 2. Fit of the measured oscillations of the current steps of point contact No. 4 (symbols) by means of the dependencies calculated from the RSJ model, reported in eq. (1) of the main paper, by using $\Omega_\varphi = 0.31$ ($I_s = I_c \sin(\varphi)$, solid blue line) and $\Omega_{2\varphi} = 0.8$ ($I_s = I_c \sin(2\varphi)$, dashed magenta line).

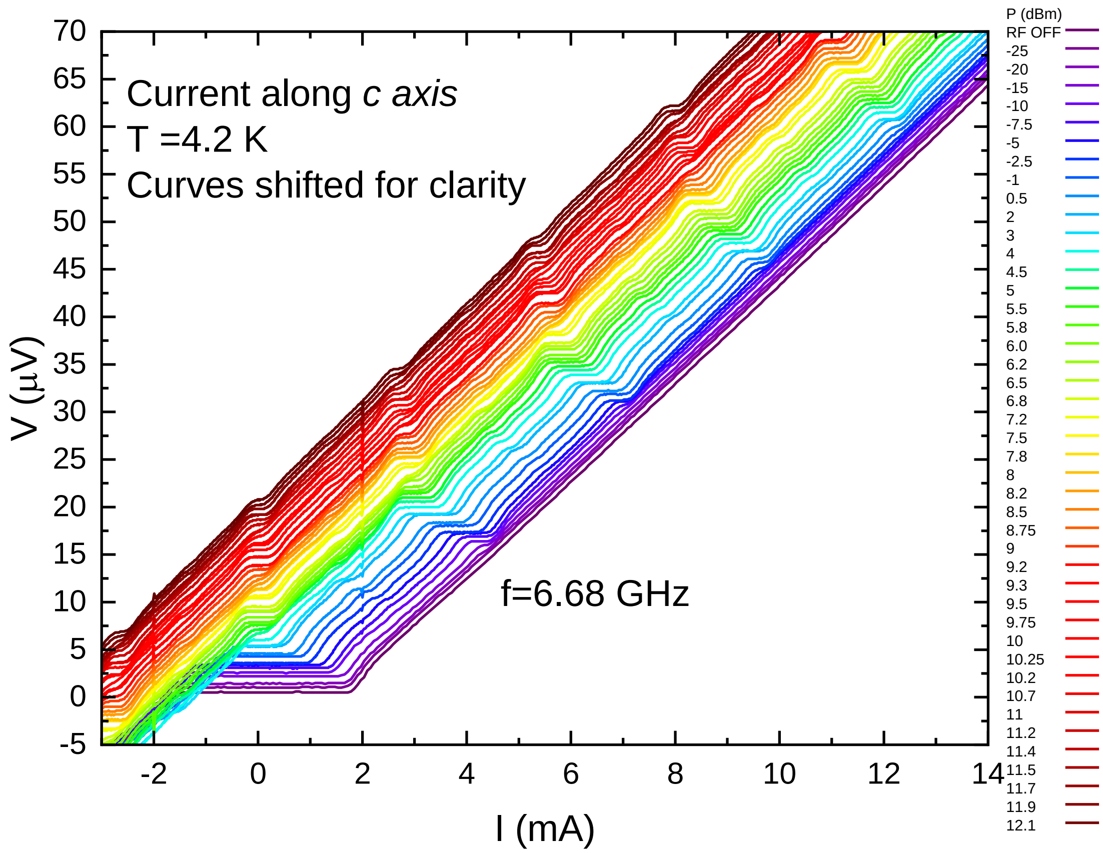

Supplementary Figure 3. Current-voltage characteristics of the $Pb_{0.6}In_{0.4}$ / $Ba_{0.6}K_{0.4}(FeAs)_2$ Josephson point-contact No. 2 recorded at *f* = 6.68 GHz and different levels of microwave power. The legend reports the attenuation of the microwave power in decibels.

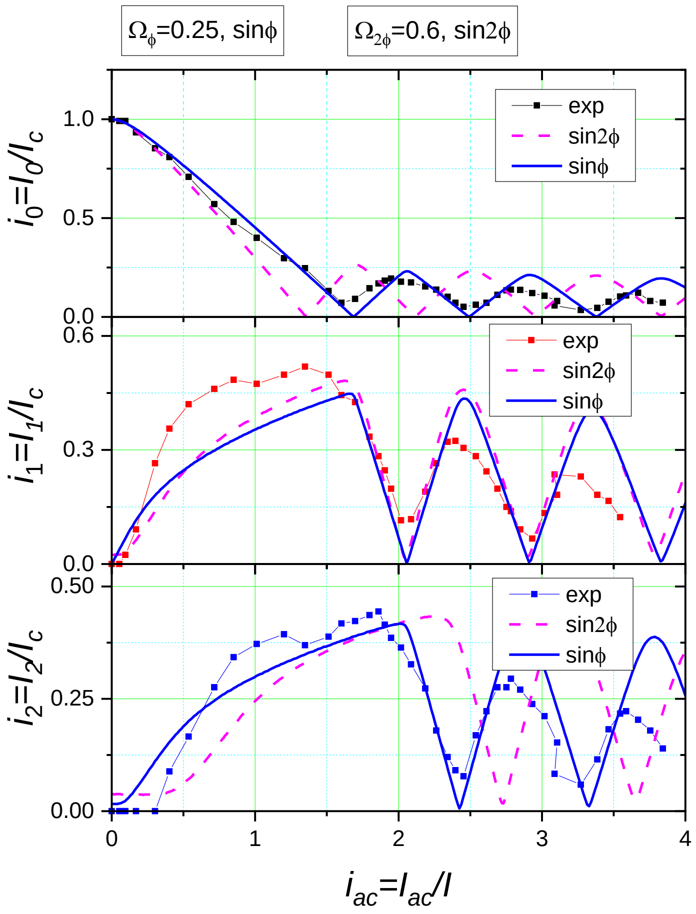

Supplementary Figure 4. Fit of the measured oscillations of the current steps of point contact No. 2 (symbols) by means of the dependencies calculated from the RSJ model, reported in eq. (1) of the main paper, by using Ω_φ = 0.25 (I_s = I_c sin(φ), solid blue line) and $\Omega_{2\varphi}$ = 0.6 (I_s = I_c sin(2φ), dashed magenta line).
